# Somatostatin neurons control an alcohol binge drinking prelimbic microcircuit in mice

**DOI:** 10.1038/s41386-021-01050-1

**Published:** 2021-06-10

**Authors:** Nigel C. Dao, Dakota F. Brockway, Malini Suresh Nair, Avery R. Sicher, Nicole A. Crowley

**Affiliations:** 1 Department of Biology, Pennsylvania State University, University Park, PA USA; 2 Neuroscience Curriculum, Pennsylvania State University, University Park, PA USA

**Keywords:** Cellular neuroscience, Addiction

## Abstract

Somatostatin (SST) neurons have been implicated in a variety of neuropsychiatric disorders such as depression and anxiety, but their role in substance use disorders, including alcohol use disorder (AUD), is not fully characterized. Here, we found that repeated cycles of alcohol binge drinking via the Drinking-in-the-Dark (DID) model led to hypoactivity of SST neurons in the prelimbic (PL) cortex by diminishing their action potential firing capacity and excitatory/inhibitory transmission dynamic. We examined their role in regulating alcohol consumption via bidirectional chemogenetic manipulation. Both hM3Dq-induced excitation and KORD-induced silencing of PL SST neurons reduced alcohol binge drinking in males and females, with no effect on sucrose consumption. Alcohol binge drinking disinhibited pyramidal neurons by augmenting SST neurons-mediated GABA release and synaptic strength onto other GABAergic populations and reducing spontaneous inhibitory transmission onto pyramidal neurons. Pyramidal neurons additionally displayed increased intrinsic excitability. Direct inhibition of PL pyramidal neurons via hM4Di was sufficient to reduce alcohol binge drinking. Together these data revealed an SST-mediated microcircuit in the PL that modulates the inhibitory dynamics of pyramidal neurons, a major source of output to subcortical targets to drive reward-seeking behaviors and emotional response.

## Introduction

Alcohol use disorder (AUD) is a chronically relapsing disorder (with three sub-classifications: mild, moderate, severe), and is characterized by the presence of a minimum of 2 symptoms related to both alcohol consumption behavior and the ramifications of that behavior [1, 2]. Binge drinking, in particular, has been shown to have severe negative health outcomes, with over 26% of adults (18 + ) reporting binge drinking in the past month [3]. The National Institute on Alcohol Abuse and Alcoholism (NIAAA) defines binge drinking as a consumption pattern leading to a blood alcohol content greater than 80 mg/dL in a 2 h period (approximately 4 drinks for women or 5 drinks for men). Though both men and women engage in binge drinking, sex differences exist in both onset and patterns of binge drinking and outcomes (for a review, see [4]).

Multiple brain regions, neuronal subtypes, neurotransmitters, and neuromodulators have been implicated in alcohol’s effects [5]. The prelimbic (PL) cortex is a key brain region in addiction and substance use [6, 7], with projections to many limbic areas [7] including the bed nucleus of the stria terminalis (BNST) [8, 9] and the nucleus accumbens [10]. These PL glutamatergic outputs are thought to provide top-down regulation of affective and motivational behaviors. In addition, the PL receives excitatory glutamate afferents from the basolateral amygdala [11, 12], and the hippocampus [13, 14], and receive internal inhibitory modulation from a variety of subpopulations of gamma-aminobutyric acid (GABA) neurons that express peptidergic markers and receptors such as dynorphin (DYN; 14), corticotropin-releasing factor (CRF; [15, 16]), neuropeptide-y (NPY; [17]) and somatostatin (SST; [18, 19]). Neurotransmission within the PL is known to be altered in multiple animal models of alcohol exposure, including binge drinking [20] and vapor ethanol exposure [21, 22]. Recent work has demonstrated that repeated cycles of binge drinking diminishes NPY expression in the PL, and that activation of NPY signaling within the PL otherwise can reduce binge drinking [17], highlighting the engagement of these peptide-expressing GABA neurons in AUD and their therapeutic potentials.

Human and preclinical rodent evidence points to SST neurons as a key modulator of neuropsychiatric disorders, in particular anxiety and depression [23]. Global genetic upregulation of SST neurons reduces anxiety-like and depression-like behavior in mice [24], with site-specific manipulations of PL SST neurons having a similar effect [25]. In addition, PL SST neurons specifically encode fear-related memories in fear conditioning [26] and social fear [27]. Despite the strong implications for SST neurons as a positive target for mood disorders, their role in AUD remains to be explored. Here, we demonstrate that PL SST neurons are strongly modulated by binge alcohol consumption in both male and female mice, and that bi-directional chemogenetic modulation of SST neurons in the PL reduces binge alcohol consumption in both sexes (with a greater overall effect in females). In addition, we provide new insight into the overall circuitry of SST neurons within the PL and SST neurons’ role in maintaining signaling of PL pyramidal output neurons.

## Methods

### Animals

A total of 138 mice were used in these experiments. All experiments were approved by the Pennsylvania State University Institutional Animal Care and Use Committee. Adult (over 8 weeks of age) male and female C57BL/6 J (stock #000664, The Jackson Laboratory), hemizygous SST-IRES-Cre mice (stock #013044, The Jackson Laboratory) and *Ai9* reporter mice (stock #007909, The Jackson Laboratory) on C57BL6/J background were bred in-house and genotyped by standard PCR protocol (additional detail available in supplemental methods).

### Surgeries

Where applicable, adult mice underwent intra-PL (from Bregma, AP: + 1.8 mm, ML: ± 0.4 mm, DV: −2.3 mm) viral injections. Mice were deeply anesthetized with isoflurane (5% induction, 1–2% maintenance) and mounted on the stereotaxic frame (Stoelting, Wood Dale, IL). Following craniotomy, 0.3 uL of virus per side was injected into the PL at a rate of 0.1 μl/min via a 1μl Hamilton Neuros Syringe (additional details, including viral vectors, available in supplemental methods and Supplementary Figure 2).

### Drinking in the Dark (DID)

DID was conducted as previously published [20, 28]. Mice received 20% (v/v) ethanol (EtOH; Koptec, Decon Labs, King of Prussia, PA) in tap water, 3 h into the dark cycle for 2 h (i.e., 10 a.m. to 12 p.m.) on three sequential days. On the fourth day, they received EtOH for 4 h (i.e., 10 a.m. to 2 p.m.). Following the binge day, mice had three days of abstinence before repeating the cycle (number of cycles indicated per experiment below). For experiments involving surgical manipulations, mice underwent a baseline week of DID before surgeries as is consistent with the literature.

### Sucrose DID

In some cohorts, four days after the last EtOH binge session, mice underwent sucrose DID conducted as previously published [17, 29]. Mice received 10% (w/v) sucrose in tap water 3 h into the dark cycle on three sequential days. On the fourth day, they received the sucrose for 4 h (making sucrose DID overall experimentally similar to alcohol DID). The mice underwent three cycles total.

### Drug administration

Clozapine n-oxide (CNO; #HB1807, HelloBio, Princeton, NJ) was dissolved to 30 mg/ml in pure DMSO (Fisher Scientific, Waltham, MA) and subsequently diluted to 0.3 mg/ml in 0.9% saline. Salvinorin B (SalB; #HB4887, HelloBio, Princeton, NJ) was dissolved to 10 mg/ml in pure DMSO. Additional details on drug administration are available in supplemental methods.

### Electrophysiology

Whole-cell voltage clamp and current clamp recordings were conducted in the PL similarly to those previously published (spontaneous excitatory and inhibitory postsynaptic currents, sEPSCs and sIPSCs respectively, and intrinsic excitability [20, 21, 30]; confirmation of DREADD expression and function [31]). Full electrophysiology experimental details are available in the supplemental methods.

sEPSCs and sIPSCs were measured in voltage-clamp (held at −55 mV and +10 mV, respectively) using electrodes filled with a cesium-methanesulfonate (Cs-Meth) intracellular recording solution (containing the following, in mM: 135 Cs-methanesulfonate, 10 KCl, 10 HEPES, 1 MgCl2, 0.2 EGTA, 4 Mg-ATP, 0.3 GTP, 20 phosphocreatine, 287–290 mOsm, pH 7.33). In some experiments, tetrodotoxin (TTX; 500 nM) was included in the aCSF to isolate miniature IPSCs (mIPSCs). Frequency (Hz) and amplitude (pA) of PSCs (both spontaneous and miniature) within individual neurons were calculated in a 2-minute epoch following a minimum of 10 min of stabilization. Measurements of intrinsic excitability were recorded in current clamp, including resting membrane potential (RMP), rheobase (the minimum amount of current needed to elicit an action potential), action potential threshold (the membrane potential at which the first action potential fired), and current-injection induced firing (0–200 pA, 10 pA per step). Experiments were performed at both RMP and the holding potential −70 mV. Electrodes were filled with a potassium gluconate-based (KGluc) intracellular recording solution (in mM: 135 K-Gluc, 5 NaCl, 2 MgCl2, 10 HEPES, 0.6 EGTA, 4 Na2ATP, and 0.4 Na2GTP, 287–290 mOsm, pH 7.35).

To confirm the functionality of the hM3Dq and KORD viruses, CNO (10 μM) or SalB (1 μM) was added to the recording aCSF for 10 min following a 10 min stabilization of RMP, and recording continued for a 15-min washout. Tetrodotoxin (500 nM) was added to the recording aCSF to block action potentials for consistent measurement of RMP. Average RMP was normalized to a 5 min baseline. To record sIPSCs on pyramidal neurons during CNO and SalB bath application, 3 mM kynurenic acid was included in the recording aCSF to block α-amino-3-hydroxy-5-methyl-4-isoxazolepropionic acid (AMPA) and N-methyl-D-aspartate (NMDA) receptor-mediated EPSCs using a potassium chloride/potassium-gluconate (KCl-KGluc) intracellular recording solution (in mM: 70 potassium gluconate, 80 KCl, 10 HEPES, 1 EGTA, 4 Na2ATP, 0.4 Na2GTP, 287–290 mOsm, pH 7.3). Neurons were held at −70 mV. After a stable 6-min baseline, CNO (10 μM) or SalB (1 μM) in aCSF+kynurenic acid was perfused onto the slice for 10 min, followed by 15 min of aCSF+kynurenic acid only washout. Frequency and amplitude of postsynaptic events were normalized to a 6-min baseline period.

To assess the local connectivity between SST neurons and both pyramidal neurons and non-SST putative GABAergic neurons, optogenetically-evoked IPSCs were elicited by 2 ×1 ms pulses of 470 nM blue light delivered 100 ms apart (Cool LED, Traverse City, MI, USA) using the K-gluc intracellular recording solution (see above).

Signals were digitized at 10 kHz and filtered at 3 kHz using a Multiclamp 700B amplifier and analyzed using Clampfit 10.7 software (Molecular Devices, Sunnyvale, CA). For all measures, recordings were performed in a maximum of two neurons per subregion, per mouse, and lowercase *n* values reported reflect the number of neurons for each measure.

### Statistical analysis

Experimenters were blinded to group (alcohol or water), and chemogenetic manipulation whenever possible. Data were analyzed by ANOVAs and post-hoc tests as appropriate and indicated for each experiment. Statistical significance threshold was set at ɑ = 0.05. Statistical analysis and graph construction was performed in Graphpad Prism 7.0, and finalized figures were constructed in Adobe Illustrator and BioRender. Data are presented as Means and Standard Error of the Mean (SEM).

## Results

### Alcohol binge-like drinking alters excitatory transmission onto, and neuronal intrinsic excitability of, PL SST neurons

**Fig. 1:**
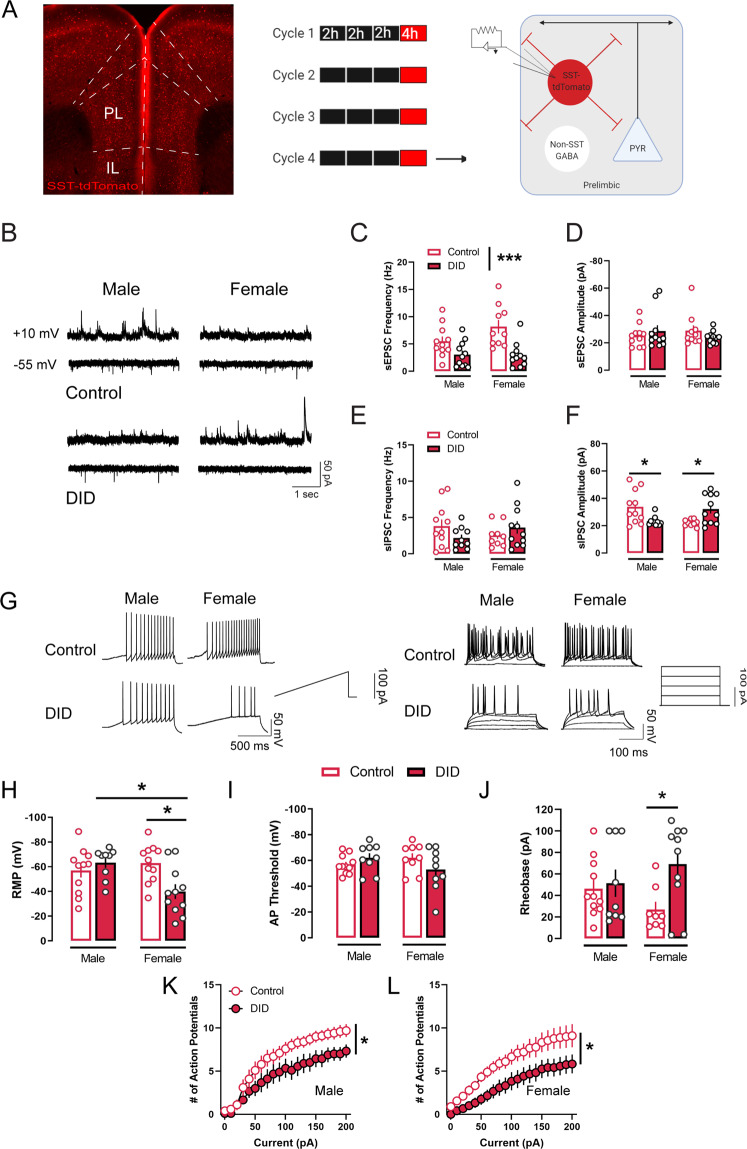
Binge-like alcohol consumption dampens intrinsic excitability and excitatory drive of SST neurons in the PL. **A** Left: PL SST neurons under tdTomato fluorescence. Right: Schematic of experimental timeline. **B** Representative sEPSC traces (top four) and sIPSC traces (bottom four) in SST cells in the PL. **C** Binge drinking reduced sEPSC frequency in SST cells in both sexes. **D** Binge drinking did not alter sEPSC amplitude in SST cells. **E** Binge drinking did not alter sIPSC frequency in SST cells. **F** sIPSC amplitude in SST cells exhibits basal sex differences, in which male SST neurons have larger inhibitory current amplitude than female SST neurons. Binge drinking sex-dependently altered sIPSC amplitude in SST cells, in which sIPSC amplitude was reduced in males, but increased in females. **G** Representative traces of rheobase and VI recording in SST cells in the PL. **H** Binge drinking depolarized resting membrane potential in female SST PL neurons, but not male. **I** Action potential threshold in SST PL neurons across sexes was not altered following binge drinking. **J** Binge drinking increased the minimum current needed to elicit an action potential in female SST PL neurons, but not males. **K** Current-induced spiking was reduced in male SST PL neurons following binge drinking. **L** Binge drinking reduced current-induced spiking in female SST PL neurons. *p < 0.05. **p < 0.01. ***p < 0.001. Bonferroni’s post-hoc test.

To examine DID-induced plasticity in PL SST neurons, *SST*-*IRES-CRE::Ai9* male and female mice underwent DID for four cycles (Fig. 1A for representative expression and experimental timeline). Similar to previous reports, female mice consumed higher levels of alcohol than male mice (Supplementary Fig. 1). 24 h after the last alcohol binge drinking session, we performed whole-cell patch clamp electrophysiology in PL SST neurons to examine DID-induced alterations in intrinsic excitability in and synaptic transmission on these neurons. First, voltage clamp experiments revealed that alcohol binge drinking disrupted the excitation/inhibition dynamics onto PL SST neurons. Both DID-exposed male and female DID exposed SST neurons showed an attenuation of sEPSC frequency as compared to their control counterparts (control male: *n* = 11 cells/5 mice, DID male: *n* = 11 cells/6 mice, control female: *n* = 10 cells/5 mice, DID female: *n* = 11 cells/ 5 mice, F_sex_(1,39) = 2.1, *p* = 0.155; F_binge_(1,39) = 18.6, *p* < 0.001, F_sex x binge_(1,39) = 2.4, *p* = 0.123; Fig. 1B, C), with no changes in sEPSC amplitude (F_sex_(1,38) = 0.0, *p* = 0.8763; F_binge_(1,39) = 0.06, *p* = 0.798, F_sex x binge_(1,39) = 1.7, *p* = 0.199; Fig. 1D). While we found no changes in sIPSC frequency across sexes and drinking groups (control male: *n* = 11 cells, DID male: *n* = 10 cells, control female: *n* = 9 cells, DID female: *n* = 11 cells; F_sex_(1,37) = 0.0, *p* = 0.884; F_binge_(1,37) = 0.1, *p* = 0.698, F_sex x binge_(1,37) = 3.2, *p* = 0.080; Fig. 1E), DID produced opposite adaptations in PL SST neuronal sIPSC amplitude between sexes (F_sex_(1,37) = 0.2, *p* = 0.62; F_binge_(1,37) = 0.0, *p* = 0.932, F_sex x binge_(1,37) = 14.1, *p* < 0.001; Fig. 1F). Whereas alcohol binge drinking reduced sIPSC amplitude in male PL SST neurons (*p* = 0.018), it otherwise augmented sIPSC amplitude in female SST neurons (*p* = 0.029). Notably, there was also a basal sex difference in control mice, where male SST neurons displayed larger sIPSC events than female SST neurons (*p* = 0.014), suggesting significant variability in the postsynaptic expression of GABA_A_ receptor between sexes that could be differentially altered by a history of binge drinking.

Additionally, current clamp experiments revealed that PL SST neurons exhibited marked changes in intrinsic excitability following binge alcohol. While there were no changes in the RMP of SST neurons in DID-exposed males, SST neurons in DID-exposed females displayed depolarized RMP compared to control females *(p* = 0.014) (control male: *n* = 11 cells/ 6 mice; DID male: 9 cells/ 5 mice; control female: 11 cells/ 5 mice; DID female: 11 cells/ 5 mice; F_sex_(1,38) = 2.7, *p* = 0.105; F_binge_(1,38) = 2.5, *p* = 0.119, F_sex x binge_(1,38) = 7.8, *p* = 0.007; Fig. 1G, H). No differences in action potential threshold of SST neurons in either sex were detected (F_sex_(1,34) = 1.1, *p* = 0.3; F_binge_(1,34) = 0.0, *p* = 0.894, F_sex x binge_(1,34) = 1.3, *p* = 0.256; Fig. 1I). Rheobase was increased in DID-exposed female SST neurons (*p* = 0.018) as compared to controls, but not in DID-exposed males (control male: *n* = 11 cells/6 mice; DID male: *n* = 9 cells/ 5 mice; control female: *n* = 8 cells/ 5 mice; DID female: *n* = 10 cells/ 5 mice; F_sex_(1,34) = 0.0, *p* = 0.941; F_binge_(1,34) = 4.9, *p* = 0.032, F_sex x binge_(1,34) = 3.0, *p* = 0.087; Fig. 1J).

In DID-exposed male SST neurons, the numbers of spikes per current step were decreased as compared to controls (control male: *n* = 10 cell/ 6 mice; DID male: *n* = 9 cells/ 5 mice; F_current_(20,340) = 71.1, *p* < 0.001; F_binge_(1,17) = 3.8, *p* = 0.061, F_current x binge_(20,340) = 1.7, *p* = 0.026; Fig. 1K), with similar results seen in DID-exposed females (control female: *n* = 9 cells/ 5 mice, DID female: *n* = 12 cells/ 5 mice, F_current_(20,380) = 39.3, *p* < 0.001; F_binge_(1,19) = 5.7, *p* = 0.027, F_current x binge_(20,380) = 0.9, *p* = 0.467).

Together, these data demonstrated that repeated cycles of alcohol binge drinking result in a state of hypoexcitability in PL SST neurons via a reduction in both excitatory synaptic transmission onto, and intrinsic excitability of these neurons, which could ultimately lead to disinhibition of neighboring populations.

### Both chemogenetic activation and inhibition of SST neuron activity reduce binge-like alcohol drinking via direct and indirect inhibition of pyramidal neurons

**Fig. 2:**
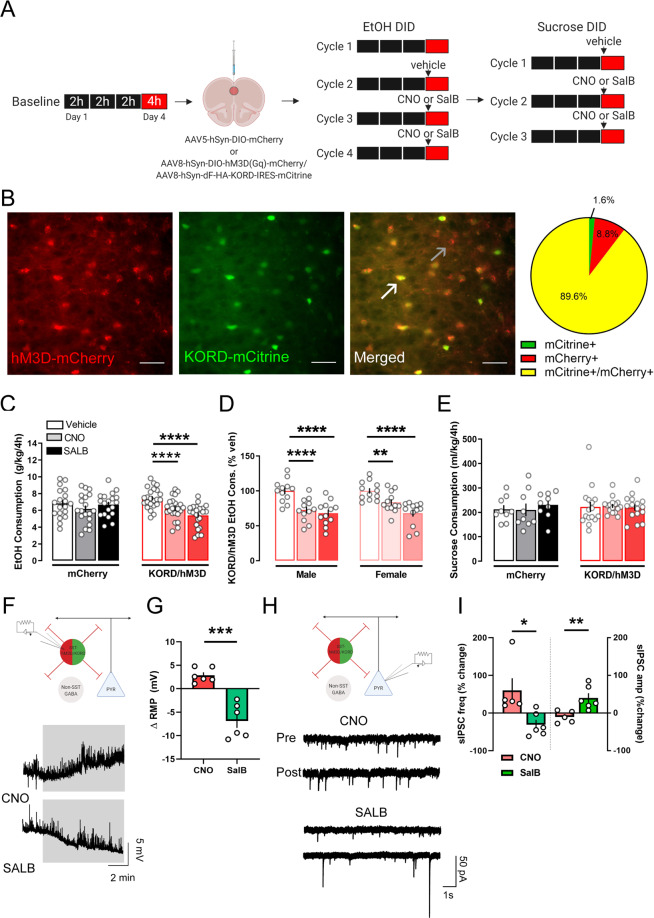
Bi-directional control of PL SST neurons reduces binge drinking. **A** Schematic of the experimental design. Both male and female SST-IRES-Cre mice were used in this experiment. **B** Representative images of hM3Dq- and KORD-expressing SST neurons at high magnification [40x]. White arrows: mCitrine + /mCherry+ cells. Black arrows: mCherry+ only cells. Scale bar 50 μM. (Right) Percent of total infected SST neurons in the PL expressing mCitrine-tagged KORD (green) only, mCherry-tagged hM3Dq only (red), or both viruses (yellow). **C** CNO-induced activation and SalB-induced silencing of SST PL neurons reduced binge drinking in hM3Dq- and KORD-expressing mice, but not in mCherry-expressing control mice. **D** In hM3Dq- and KORD-expressing mice, females and males were equally susceptible to activation and silencing of SST PL neurons. **E** Manipulation of SST PL neurons did not affect sucrose binge drinking. **F** Schematic of electrophysiology recording in DREADD-expressing SST neurons and representative traces of RMP of hM3Dq- and KORD-expressing SST neurons during bath application of 10 uM CNO (top) and 1 uM SalB (bottom). **G** CNO depolarized and SalB hyperpolarized resting membrane potential of KORD- and hM3Dq-expressing SST neurons. **H** Schematic of electrophysiology recording in L2/3 pyramidal neurons and representative traces of sIPSC recordings in glutamate receptor blocker (3 mM kynurenic acid)-containing aCSF before and after bath application of 10 μM CNO and 1 μM SalB. **I** CNO-induced activation of PL SST neurons increased sIPSC frequency on pyramidal neurons with no changes in amplitude. SalB-induced inhibition of PL SST neurons otherwise produced a decrease in sIPSC frequency and an increase in sIPSC amplitude. *p < 0.05. **p < 0.01, ***p < 0.001, ****p < 0.0001. Bonferroni’s post-hoc tests.

Given previous reports on the resiliency-conferring role of PL SST neurons in affective disorders [23, 24] and our observation of a reduction in SST neuronal excitability following binge drinking, we investigated the ability of chemogenetic manipulation of PL SST neurons to alter drinking. We injected SST-IRES-Cre male and female mice with a cocktail of AAVs encoding the excitatory Gq-coupled hM3Dq and the inhibitory Gi-coupled kappa-opioid-derived DREADD (KORD) into the PL, which allowed for bidirectional manipulation of the same SST neurons during DID [31] (for experimental timeline, see Fig. 2A, representative viral injections, Fig. 2B, and Supplementary Figure 1 for quantification of overlap between hM3Dq and KORD**)**.

Both activation and inhibition of PL SST neurons (via CNO and SalB systemic administration, respectively) reduced alcohol consumption during the binge sessions in hM3Dq/KORD-expressing mice (*N* = 24; 12 males and 12 females) (Vehicle vs. CNO: *p* < 0.001; Vehicle vs. SalB: *p* < 0.001; F_drug_(2,80) = 17.1, *p* < 0.001, F_virus_(1,40) = 0.3, *p* = 0.593, F_virus x drug_(2,80) = 11.8, *p* < 0.001, Fig. 2C), with no changes in alcohol drinking in viral control-injected mice (*N* = 19; 10 males and 9 females) This effect was seen in both sexes of hM3Dq/KORD-expressing mice (F_drug_(1,22) = 0.4, *p* = 0.494, F_sex_(2,44) = 49.1, *p* < 0.001, F_sex x drug_(2,44) = 1.8, *p* = 0.168; Fig. 2D). Consumption of 10% sucrose DID (Fig. 2E) was unaltered in DREADD-expressing and control mice (F_drug_(2,46) = 0.1, *p* = 0.85, F_virus_(1,23) = 0.1, *p* = 0.768, F_virus x drug_(2,46) = 0.4, *p* = 0.669), suggesting that the CNO- and SalB-induced effect was specific to alcohol consumption in DREADD-expressing mice, and not generalizable to other rewarding fluids.

Next, we performed whole cell patch clamp on hM3Dq/KORD-expressing SST neurons, visualized by mCherry and mCitrine fluorescence respectively, to verify the functionality of the DREADDs. As expected, a 10 min bath application of CNO produced a significant depolarization in the RMP of SST neurons (baseline vs. washout, paired *t*(5) = 3.2, *p* = 0.022), whereas SalB resulted in a significant hyperpolarization (paired *t*(6) = 4.4, *p* = 0.007; CNO vs. SalB unpaired *t*(10) = 5.4, *p* < 0.001; Fig. 2F, G). Voltage clamp experiments on layer 2/3 pyramidal neurons revealed an increase in sIPSC frequency following bath CNO application (baseline vs. washout, paired *t*(5) = 4.0, *p* = 0.016), with no change in amplitude (paired *t*(5) = 1.1, *p* = 0.325; Fig. 2H, I). Following bath application of SalB there was a decrease in sIPSC frequency (paired *t*(6) = 2.7, *p* = 0.039), and an increase in sIPSC amplitude (paired *t*(7) = 5.6, *p* = 0.002; Fig. 2I) – suggesting a more nuanced microcircuitry may be mediating the curious bidirectional behavioral effects.

Overall, these data revealed the role of PL SST neurons in regulating binge-like alcohol intake by regulating the inhibitory dynamics onto pyramidal neurons, the main source of excitatory output from the PL cortex.

### A sex-specific SST-mediated inhibitory circuit in the PL underlies DID-induced adaptations

**Fig. 3:**
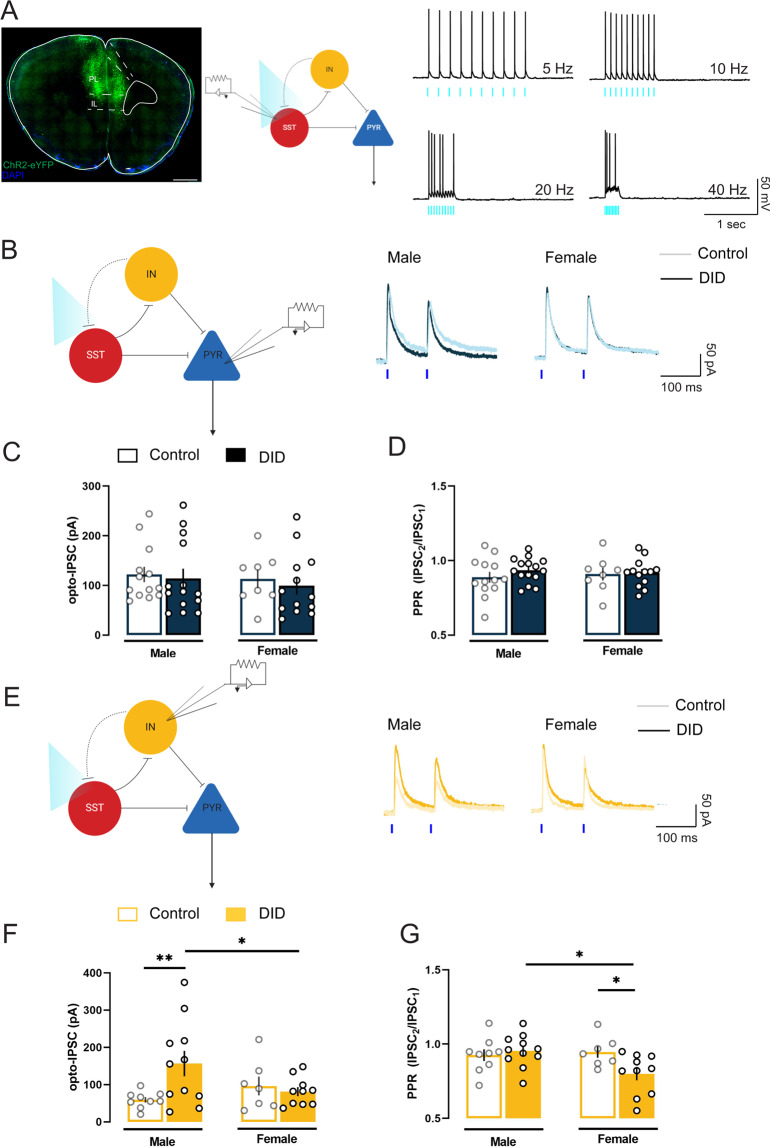
SST-mediated inhibitory dynamic in the PL is moderated by sex and alcohol binge drinking. **A** (Left) ChR2-expressing SST neurons in the PL. Scale bar 1 mm. (Right) Photostimulated action potential fidelity in SST neurons upward to 10 Hz. **B** Representative traces of SST-mediated IPSCs in PL pyramidal neurons in males and females. **C** Binge drinking did not alter SST-mediated IPSC amplitude in PL pyramidal neurons in either sex. **D** No differences in SST-evoked IPSC paired pulse ratio on pyramidal neurons across groups and sexes. **E** Representative traces of SST-mediated IPSCs in PL non-SST, putatively GABAergic neurons in males and females. **F** Binge drinking enhanced SST-mediated IPSC amplitude onto non-SST neurons in male mice, but not female mice. **G** Binge drinking reduced SST-mediated IPSC paired pulse ratio in female mice, but not male mice. DID: *N* = 5 male mice and 5 female mice, control: *N* = 6 male mice and 4 female mice. *p < 0.05. **p < 0.01. Bonferroni’s post-hoc test.

Previous work in fear conditioning [26], social defeat stress [27], and morphine exposure [32] in male mice suggest that PL SST neurons interact with an intermediary GABAergic source to disinhibit pyramidal neurons and heighten stress response and reward-seeking. To examine the plasticity adaptations following alcohol binge drinking in the SST neuron-mediated inhibitory circuit in the PL, we injected SST-IRES-Cre male and female mice with the Cre-dependent viral construct encoding for the light-inducible channelrhodopsin-2 (ChR2) in the PL (Fig. 3A). Action potentials in ChR2+ SST neurons were elicited by 1 ms blue light (470 nm) photostimulation, with full action potential fidelity upward of 10 Hz (Fig. 3A), consistent with SST firing patterns we have previously published [33]. Next, we used a paired-pulse photostimulation protocol (2 ×1 ms pulses, 100 ms interval) to evoke release from SST neurons while recording from either pyramidal neurons or putatively GABAergic, non-SST neurons. The cell-type identity was confirmed by membrane properties (capacitance and membrane resistance, described in the methods and shown in Supplementary Fig. 3D, E) and action potential characteristics (Supplementary Fig. 3F, G), which was consistent with the published literature [26]. This stimulation protocol revealed direct, monosynaptic connections between SST neurons and both neighboring subpopulations, in which photostimulation of SST neurons induced potent, time-locked IPSCs in both pyramidal neurons and non-SST non-PYR (putative GABAergic) neurons (Supplementary Fig. 3A–C).

Alcohol binge drinking did not alter SST-evoked IPSC amplitude onto pyramidal neurons either both males or females (control male: *n* = 13 cells/ 5 mice; DID male: *n* = 14 cells/ 5 mice; control female: *n* = 8 cells/ 5 mice; DID female: *n* = 13 cells/ 5 mice; F_sex_(1,44) = 0.4, *p* = 0.526, F_binge_(1,44) = 0.3, *p* = 0.571, F_sex_ _x binge_(1,44) = 0.0, *p* = 0.891; Fig. 3B, C). IPSC paired pulse ratio (PPR), a measurement of presynaptic GABA release probability, was also unaltered in pyramidal neurons of DID-exposed mice as compared to controls (F_sex_(1,44) = 0.0, *p* = 0.959, F_binge_(1,44) = 0.7, *p* = 0.388, F_sex x binge_(1,44) = 0.4, *p* = 0.522, Fig. 3D). Alcohol binge drinking, however, enhanced SST-evoked inhibitory transmission onto other non-SST, GABAergic populations in a sex-dependent manner. SST-evoked IPSC amplitude onto non-SST neurons was increased in DID-exposed male mice as compared to control males (*p* = 0.009) and DID-exposed female (*p* = 0.044) (control male: *n* = 9 cells/ 5 mice; DID male: *n* = 11 cells/ 5 mice; control female: 7 cells/ 5 mice; DID female: *n* = 10 cells/ 5 mice; F_sex_(1,33) = 0.6, *p* = 0.433, F_binge_(1,33) = 2.9, *p* = 0.094, F_sex x binge_(1,33) = 5.5, *p* = 0.025, Fig. 3E, F). IPSC PPR was decreased in non-SST neurons of DID-exposed females as compared to control females (*p* = 0.033) and DID-exposed males (*p* = 0.010) (F_sex_(1,33) = 2.8, *p* = 0.099, F_binge_(1,33) = 2.2, *p* = 0.143, F_sex x binge_(1,33) = 4.9, *p* = 0.032, Fig. 3G).

Together, these data provided evidence for a microcircuit of SST neurons in the PL to, directly and indirectly, gate the gain of pyramidal neurons to control binge-like alcohol drinking, with sex differences in the relative strength of SST neuron-mediated inhibition of excitatory pyramidal neurons and other GABAergic populations. Repeated cycles of excessive alcohol use disrupted these dynamics and disinhibited pyramidal neurons in the PL by enhancing SST-mediated inhibitory tone onto other GABAergic sources.

### Direct inhibition of PL pyramidal neurons reduces binge-like alcohol drinking

**Fig. 4:**
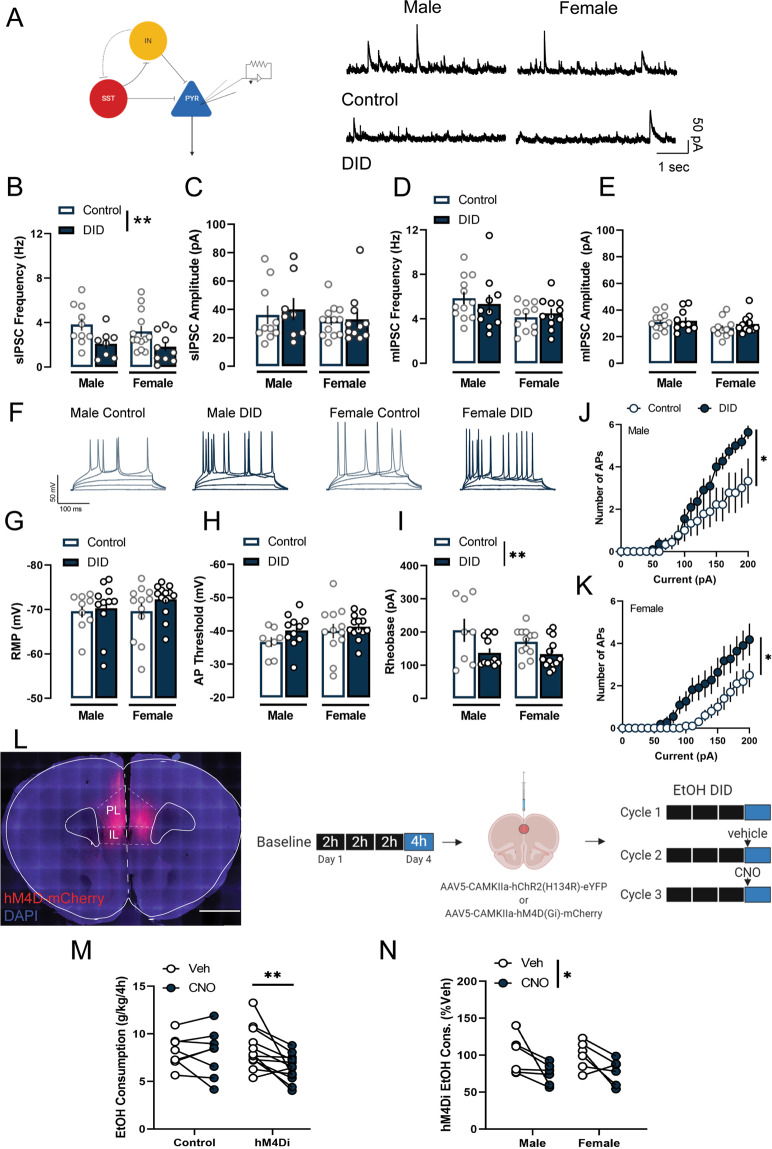
Alcohol binge drinking disinhibits pyramidal neurons and increases pyramidal intrinsic excitability, while chemogenetic silencing of pyramidal neurons reduces alcohol consumption. **A** Representative sIPSC traces in PL L2/3 pyramidal neurons in control and binge drinking mice in both sexes. **B** Binge drinking reduced sIPSC frequency in pyramidal neurons. **C** Binge drinking did not alter sIPSC amplitude in pyramidal neurons. **D** No alterations in mIPSC frequency in pyramidal neurons. **E** No alterations in mIPSC amplitude in pyramidal neurons. **F** Representative traces of action potentials per current step in pyramidal neurons in control and binge drinking mice in both sexes. **G** Binge drinking did not alter RMP in either sex. **H** Binge drinking did not alter action potential threshold in either sex. **I** Pyramidal neurons in both sexes had lower rheobase following binge drinking. **J** Male pyramidal neurons produced more action potentials with increasing current injection amplitude following binge drinking. **K** Female pyramidal neurons produced more action potentials with increasing current injection amplitude following binge drinking. **L** Representative images of the hM4D(Gi) viral expression in PL pyramidal neurons. Scale bar 1 mm. (Right) Schematic of the experimental design. Both male and female C57BL/6 J mice were used in this experiment. **M** CNO-induced inhibition of PL pyramidal neurons decreased binge drinking in hM4D(Gi)-expressing mice. **N** Inhibition of PL pyramidal neurons was equally effective in reducing binge drinking in both male and female mice. *p < 0.05. **p < 0.01. Bonferroni’s post-hoc test.

Given that pyramidal neurons are the major output source of the PL to subcortical targets, including the striatum, the amygdala and the midbrain to drive reward-seeking behaviors and emotional responses, we next investigated the effects of alcohol binge drinking on the inhibitory dynamics onto PL pyramidal neurons. There was a reduction in sIPSC frequency on L2/3 pyramidal neurons alcohol following binge drinking in both sexes (male control: *n* = 10 cells/ 6 mice, male DID: *n* = 8 cells/ 5 mice, female control: *n* = 13 cells/ 5 mice, female DID: *n* = 10 cells/ 5 mice; (F_sex_(1,37) = 0.7, *p* = 0.381; F_binge_(1,37) = 9.5, *p* = 0.003, F_sex x binge_(1,37) = 0.1, *p* = 0.718, Fig. 4A, B), with no change in sIPSC amplitude (F_sex_(1,37) = 1.0, *p* = 0.31; F_binge_(1,37) = 0.1, *p* = 0.657, F_sex x binge_(1,37) = 0.0, *p* = 0.798, Fig. 4C). In the presence of the voltage-gated sodium channel blocker TTX, there was no change in either frequency or amplitude of mIPSC (control male: *n* = 12 cells/ 5 mice; DID male: *n* = 10 cells/ 5 mice; control female: *n* = 11 cells/ 5 mice; DID female: *n* = 11 cells/ 5 mice; frequency: F_sex_(1,40) = 5.3, *p* = 0.026; F_binge_(1,40) = 0.0, *p* = 0.897, F_sex x binge_(1,40) = 0.6, *p* = 0.433, Fig. 4D; amplitude: F_sex_(1,40) = 2.6, *p* = 0.112; F_binge_(1,40) = 0.2, *p* = 0.601, F_sex x binge_(1,40) = 0.9, *p* = 0.351, Fig. 4E), suggesting that the effects of alcohol binge drinking on inhibitory transmission are action potential-dependent.

In addition, alcohol binge drinking enhanced the intrinsic excitability of PL L2/3 pyramidal neurons. There was no changes in RMP (control male: *n* = 8 cells/5 mice; DID male: *n* = 11 cells/5 mice, control female: *n* = 12 cells/5 mice; DID female: *n* = 13 cells/5 mice; F_sex_(1,40) = 0.5, *p* = 0.463; F_binge_(1,40) = 1.4, *p* = 0.242, F_sex x binge_(1,40) = 0.2, *p* = 0.627, Fig. 4G), or action potential threshold (F_sex_(1,40) = 1.7, *p* = 0.189; F_binge_(1,40) = 2.1, *p* = 0.152, F_sex x binge_(1,40) = 0.4, *p* = 0.498, Fig. 4H). However, alcohol binge drinking reduced the rheobase in both sexes (F_sex_(1,40) = 1.2, *p* = 0.275; F_binge_(1,40) = 9.0, *p* = 0.005, F_sex x binge_(1,40) = 0.7, *p* = 0.381, Fig. 4I). In addition, the number of action potentials per current steps was also increased in DID-exposed mice (male: F_current_(20, 360) = 49.2, *p* < 0.001; F_binge_(1,18) = 2.9, *p* = 0.104, F_sex x binge_(20,360) = 3.8, *p* < 0.001, Fig. 4J; female: F_current_(20, 380) = 30.1, *p* < 0.001; F_binge_(1,19) = 4.6, *p* = 0.044, F_sex x binge_(20,380) = 3.2, *p* < 0.001, Fig. 4K).

In line with the hyperexcitablity of pyramidal neurons following alcohol binge drinking, chemogenetic silencing of these neurons was sufficient to reduce alcohol consumption levels (Fig. 4L for representative image and experimental timeline). We injected wildtype C57Bl6/J males and females in the PL with a viral construct encoding for the inhibitory Gi-coupled hM4D DREADD under the control of the CamKIIα promoter. PL pyramidal silencing decreased alcohol intake (F_drug_(1,18) = 5.7, *p* = 0.028, F_virus_(1,18) = 0.6, *p* = 0.434, F_virus x drug_(1,18) = 4.2, *p* = 0.050, hM4Di vehicle vs. CNO: *p* = 0.005, control vehicle vs. CNO: *p* > 0.99; Fig. 4M). There was no sex difference in responsiveness to pyramidal inhibition (F_drug_(1,10) = 9.8, *p* = 0.01, F_sex_(1,10) = 0.0, *p* = 0.858, F_sex x drug_(1,10) = 0.0, *p* = 0.843, Fig. 4N).

In sum, these data supported the overall framework of the PL as a critical hub for controlling alcohol consumption. Disruption of the excitation/inhibition dynamics within this microcircuitry can enhance susceptibility to binge-like alcohol drinking, and subsequent dependence and use.

## Discussion

**Fig. 5:**
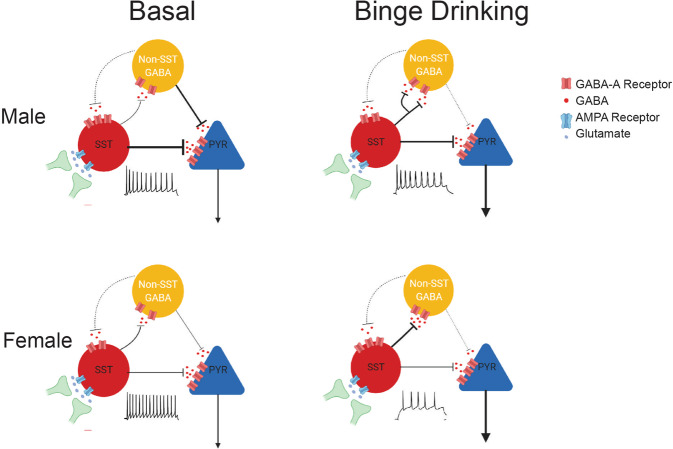
Microcircuitry of the PL cortex and binge drinking-induced plasticity. In basal states, males and females exhibit differences in spontaneous inhibitory transmission onto PL SST neurons, with higher sIPSC amplitude in males. Alcohol binge drinking disrupts the excitation/inhibition dynamic on SST neurons by dampening excitatory transmission and reversing the sex difference in inhibitory transmission. Concurrently, alcohol binge drinking dampens the intrinsic excitability of PL SST neurons, resulting in a hypoactive state. Alcohol binge augments SST neuron inhibitory output onto other GABAergic populations. Pyramidal inhibition is critical for curbing alcohol consumption behaviors, thus disinhibition of pyramidal and hyperexcitability could increase the risk of compulsive alcohol drinking and alcohol dependence.

Here, we demonstrate evidence for the role of SST neurons in the PL cortex in binge-like alcohol consumption. Our data expand upon previous reports on the involvement of this GABAergic population in a host of neuropsychiatric disorders, particularly depression [23, 24] and anxiety [25]. Repeated cycles of alcohol binge drinking altered intrinsic excitability and excitation/inhibition dynamics of PL SST neurons, resulting in a state of hypoactivity within this population (Fig. 1). Bidirectional manipulation of PL SST neurons via co-expressed excitatory and inhibitory DREADDs paradoxically reduced alcohol consumption following both excitation and inhibition of these neurons (Figs. 2 and 3). Alcohol binge drinking augmented the inhibitory strength of SST neurons onto other non-SST neurons (Fig. 3), thereby disinhibiting pyramidal neurons via decreased inhibitory transmission and increased intrinsic excitability (Fig. 4). Lastly, facilitating pyramidal inhibition could mimic the effect of SST neuron manipulation in reducing binge drinking, providing a secondary approach for reducing alcohol consumption via this circuit (Fig. 4). Overall, these results confirm and expand upon the role of the PL cortex as a critical neural hub for the development of excessive alcohol consumption, and SST neurons as a key regulator of local PL network activity (see Fig. 5 for a model of binge drinking-induced adaptations in the SST-mediated inhibitory circuit in the PL).

### Neuroadaptations in PL L2/3 pyramidal neurons

Previous reports have demonstrated the multitude of neural adaptations in PFC pyramidal neurons following alcohol dependence and withdrawal. Male C57BL6/J mice exposed to the chronic intermittent ethanol vapor (CIE) model of alcohol dependence exhibit elevated intrinsic excitability in layer 2/3 pyramidal neurons [21, 22], increased dendritic spine density [22, 34], and upregulated NMDA receptor expression [34]. Intragastric administration of high ethanol doses in rats (5 g/kg) diminishes GABA_A_ receptor-mediated inhibitory currents in PFC layer 5/6 pyramidal neurons accompanied by a reduction in expression of the α1 GABA_A_ receptor subunit [35]. Previously, we demonstrated that four cycles of DID reduces expression of cell-surface GluA2/3 and GluN1 receptors in the PL and reduced sEPSC frequency in L2/3 pyramidal neurons [20]. Additionally, female mice show distinct susceptibility to the effects of the fast-acting antidepressant ketamine in normalizing binge drinking-induced alterations in glutamatergic transmission that is not evident in male mice [20]. Here we found that in addition to its effects on glutamatergic transmission in pyramidal neurons, binge drinking further disrupted the excitation/inhibition dynamics in PL pyramidal neurons via reduced spontaneous inhibitory transmission and enhanced intrinsic excitability of these neurons. The alterations in inhibitory transmission were abolished in the presence of tetrodotoxin, suggesting an action potential-dependent mechanism. Furthermore, there is evidence that the effects of alcohol consumption on synaptic plasticity might be projection target-dependent. Excitatory synaptic strength is heightened following intermittent-access alcohol drinking in L5/6 intratelencephalic pyramidal neurons without changes in extratelencephalic neurons [36]. In addition to their distinct anatomical distribution, molecular profiles, and electrophysiological properties [36–39], these two populations are differentially involved in reward-seeking processes of natural rewards and drugs of abuse [40–42]. Nucleus accumbens (NAC)-projecting pyramidal neurons display excitation in response to sucrose-predictive cues and cocaine exposure [40, 41], and PFC input to NAC is necessary for reinstatement of cocaine-seeking behaviors [43, 44]. In contrast, paraventricular thalamus (PVT)-projecting neurons [45] and periaqueductal gray (PAG)-projecting neurons in L5/6 display inhibition in response to sucrose-predictive cues [41] and alcohol in compulsive-like drinking mice [45], respectively. Photoinhibition of PFC terminals in PAG is sufficient to drive punishment-resistant alcohol drinking in male mice [45]. The binge drinking-induced disinhibition and hyperexcitability state of pyramidal neurons in layer 2/3, where NAC-projecting neurons are most abundant [37], might further drive excessive alcohol consumption by augmenting cortical output to the NAC (Fig. 5). In line with this framework, chemogenetic silencing of pyramidal neurons via hM4Di was sufficient to decrease alcohol binge drinking in both sexes.

### Neuroadaptations in PL SST neurons

We also observed that the effects of DID on neuronal intrinsic excitability and synaptic transmission in the PL extends to SST neurons, in which SST neurons displayed a state of hypoactivity with diminished intrinsic excitability and a disrupted excitatory/inhibitory transmission dynamic in both sexes. Similar effects were observed in an intermittent-access model of alcohol drinking [46]. Of note, despite a reduction in sEPSC frequency on SST neurons in both sexes, there is an increase in evoked glutamate release probability, as evidenced by reduced EPSC paired-pulse ratio [46], suggesting that there could be a compensatory mechanism on a whole-circuit level to counteract the effects on excitatory transmission in single neurons. Alterations in sEPSC frequency and glutamate release probability [46] indicate the effects of DID on a presynaptic locus, which could arise from a wide array of glutamatergic sources such as the basolateral amygdala (BLA), the ventral hippocampus, the thalamus as well as within the medial prefrontal cortex itself [12, 47, 48]. Inputs from the BLA, a key regulator of behavioral stress response, to PL pyramidal neurons drive anxiety-like behaviors in male C57BL/6 J mice, and chronic stress can potentiate BLA-to-PFC synapses [11, 49]. The infralimbic cortex (IL) and the thalamus have been implicated in the extinction of drug-seeking behavior [50] and response to alcohol cues [48], respectively. Further investigation into the effects of DID on excitatory transmission onto PL SST neurons at a synapse-specific level is needed to fully reveal how alcohol binge drinking-mediated dysregulation of glutamatergic inputs in PL SST neurons drives withdrawal-related negative affect related behaviors and escalation in drinking.

The altered excitatory synaptic inputs were accompanied by sex-dependently opposing effects of alcohol binge drinking on inhibitory transmission onto PL SST neurons. We observed a basal sex difference in phasic sIPSC amplitude in PL SST neurons of non-drinking mice. Following DID, sIPSC amplitude was enhanced in females yet reduced in male SST neurons. These data overall suggest a substantial variability in the GABA_A_ receptor expression in these neurons, which interacts with alcohol binge drinking in a sex-dependent manner.

We previously reported that PL SST neurons are hyperexcitable following prolonged withdrawal from alcohol drinking (>4 weeks) in a 2-bottle choice model [30], which contrasts with the hypoexcitable state in acute withdrawal (24-h) from DID observed here. It should be noted that the excitability recording in the former report was done following an acute stressor (forced swim). Other stress paradigms, such as fear conditioning [26] and morphine withdrawal [32], could augment excitatory drive in PL SST neurons. These findings together suggest an extensive capacity for plasticity in these neurons that could be moderated by alcohol withdrawal stage and stress, a major predictor for relapse in alcohol drinking. The cellular and molecular mechanism underlying these alterations in SST intrinsic excitability are still unclear. Ethanol exposure has been shown to modify the expression of key modulators of neuronal excitability, including ionotropic glutamate receptors [20, 34], inward-rectifying potassium channel [51], L-type voltage-gated calcium channels [52], and hyperpolarization-activated cation channels (HCN; [53]. Intermittent access to alcohol increased medium afterhyperpolarization in PL SST neurons [46], which might be mediated by M-type potassium channels and HCN channels [54]. Notably, in females following DID, PL SST neurons displayed highly depolarized resting membrane potential yet could not efficiently produce action potentials, suggesting a state of depolarization block. One hypothesis is that DID disrupts potassium exchange within these neurons via downregulation of potassium channel function, which has been observed in other GABAergic populations [51] and leads to deficits in repolarization and action potential firings. These phenomena merit further investigation to fully demonstrate the alcohol binge drinking-induced cellular and molecular adaptations.

Additionally, we observed that DID reversed this inhibition dynamic and disinhibits pyramidal neurons in the PL by augmenting SST neuron-mediated inhibition onto other putatively GABAergic populations. Similar disinhibitory effects have been observed following fear conditioning [26], social fear [27], and morphine withdrawal [32], in which SST neurons-mediated IPSC amplitude onto parvalbumin neurons is enhanced with a reduction in IPSC PPR. This effect is accompanied by either an increase in action potential firing or excitatory inputs in PL SST neurons [26, 32]. Interestingly, our optogenetic-assisted circuit mapping results, combined with our data on attenuated action potential firing and sEPSC frequency in PL SST neurons, suggested that alcohol may produce complex changes in the PL cortex that must be further teased apart. One interpretation is that the population-wide, optically-evoked IPSC transmission from SST neurons (Fig. 3) does not reflect the changes on a single-neuron level in the intrinsic excitability experiments (Fig. 1). Population-wide increase in SST-mediated inhibitory outputs may occur to compensate for the reduction in excitation of single neurons, which bears resemblance to the discrepancy between spontaneous and evoked EPSC on SST neurons in the intermittent-access model [46]. In addition, assessments of raw optogenetically evoked IPSC amplitudes must be interpreted with caution, as mouse-to-mouse variability in ChR2 injection may drive changes in overall results.

### Chemogenetic manipulation of PL SST neurons to regulate alcohol binge drinking

A major and unexpected finding in our study is that bidirectional manipulation of SST neurons activity via both excitatory and inhibitory DREADDs similarly reduced alcohol binge drinking. CNO-mediated activation of SST neurons increased sIPSC frequency onto pyramidal neurons, concordant with a direct, monosynaptic SST to pyramidal neuron connection. On the other hand, SalB-induced silencing of SST neurons decreased sIPSC frequency, while also increased sIPSC amplitude onto pyramidal neurons. Our current hypothesis is [1] CNO-induced activation of SST neurons increases inhibitory transmission onto pyramidal neurons via direct SST-to-pyramidal connection, and [2] DREADD-induced silencing of SST neurons leads to disinhibition of another local GABAergic source to maintain overall pyramidal inhibition, as evidenced by the increase in sIPSC amplitude in pyramidal neurons. The intermediate GABAergic population could include, but not limited to, parvalbumin-expressing (PV + ) neurons, which comprise the majority of cortical interneurons and provide powerful inhibition of pyramidal output in the PL [26]. Photostimulation of PV+ neurons in the PL can facilitate extinction of reward-seeking behavior [55]. Further examination is required to establish the direct involvement of PV+ neurons and/or other GABAergic populations in the SST-mediated disinhibitory circuit in PL. There are three caveats for this interpretation about the effects of chemogenetic modulations of SST neurons in the PL circuit. First, as discussed above, PL pyramidal neurons differentially encode behavioral responses to reward cues depending on their projection target. Chemogenetic activation and silencing of PL SST neurons therefore might reduce alcohol consumption via different synapses onto projection-specific pyramidal neurons. Future studies will need to examine whether SST-mediated inhibition is biased towards specific pyramidal populations. Secondly, we cannot fully rule out an uncharacterized pharmacological action of CNO and Salvinorin B in the PL circuit. In the BNST, hM3Dq-induced activation [56] and other forms of Gq-coupled signaling [57, 58] in vGAT-expressing neurons produces long-term depression of evoked EPSC in a cannabinoid receptor 1-dependent manner, suggesting the involvement of other signaling complexes beyond GABA-mediated inhibition. Finally, the differences in the administration route and delivery vehicles of CNO (intraperitoneal injection, in 1% DMSO) and SalB (subcutaneous injection, in pure DMSO) have been found to produce opposite behavioral effects in mice, including nociception, inflammation response and locomotor activity [59]. However, as we did not observe differences in alcohol and sucrose DID consumption in mCherry-expressing control mice following systemic administration of CNO and SalB, the effects of administration routes and delivery vehicles on alcohol binge drinking are likely minimal. For this reason, we did not statistically compare these two different administration routes.

While our data demonstrate the extensive interaction between PL SST neurons and alcohol binge drinking, the current study has yet to address the involvement of neuropeptide signaling, especially that of somatostatin, in modulating alcohol consumption. Here we did not assess whether the effects of hM3Dq-induced excitation of PL SST neurons on alcohol binge drinking could be partially attributed to SST peptide release and SST signaling in the PL. The neuropeptide SST has been shown to act largely via a family of Gi/o-coupled receptors (SSTR1–5) to inhibit neuronal excitability [18, 19]. Future studies should examine the effects of alcohol binge drinking on somatostatin signaling in the PL, and the potential of somatostatin peptide as a therapeutic candidate for excessive alcohol consumption.

In conclusion, these results posit prelimbic SST neurons as a potential neural substrate of binge drinking. These neurons are well-situated to exert inhibition over the local PL circuitry, thereby controlling the flow of input and output signals of this region. We identified a host of binge drinking-induced plasticity in both SST neurons and pyramidal neurons that may drive further alterations in downstream regions heavily involved in reward-seeking and emotional behaviors.

## Supplementary information

Supplementary Material

## References

[1] National Institute of Alcohol Abuse and Alcoholism. Drinking Levels Defined. National Institute of Alcohol Abuse and Alcoholism. https://www.niaaa.nih.gov/alcohol-health/overview-alcohol-consumption/moderate-binge-drinking (2020).

[2] American Psychiatric Association. Diagnostic and Statistical Manual of Mental Disorders, 5th. Ed. American Psychiatric Publishing (2013).

[3] National Institute of Alcohol Abuse and Alcoholism. Alcohol Facts and Statistics. NIAAA [WWW Document]. https://www.niaaa.nih.gov/publications/brochures-and-fact-sheets/alcohol-facts-and-statistics (2020).

[4] Wilsnack RW, Wilsnack SC, Gmel G, Kantor LW (2017). Gender differences in binge drinking prevalence, predictors, and consequences. Alcohol Res Curr Rev.

[5] Abrahao KP, Salinas AG, Lovinger DM (2017). Alcohol and the brain: neuronal molecular targets, synapses, and circuits. Neuron.

[6] Koob GF, Volkow ND (2016). Neurobiology of addiction: a neurocircuitry analysis. Lancet Psychiatry.

[7] George O, Koob GF (2010). Individual differences in prefrontal cortex function and the transition from drug use to drug dependence. Neurosci Biobehav Rev.

[8] Crowley NA, Bloodgood DW, Hardaway JA, Kendra AM, McCall JG, Al-Hasani R (2016). Dynorphin controls the gain of an amygdalar anxiety circuit. Cell Rep.

[9] Hwa LS, Neira S, Flanigan ME, Stanhope CM, Pina MM, Pati D (2020). Alcohol drinking alters stress response to predator odor via bnst kappa opioid receptor signaling in male mice. ELife.

[10] Britt JP, Benaliouad F, McDevitt RA, Stuber GD, Wise RA, Bonci A (2012). Synaptic and behavioral profile of multiple glutamatergic inputs to the nucleus cccumbens. Neuron.

[11] Lowery-Gionta EG, Crowley NA, Bukalo O, Silverstein S, Holmes A, Kash TL (2018). Chronic stress dysregulates amygdalar output to the prefrontal cortex. Neuropharmacology.

[12] McGarry LM, Carter AG (2016). Inhibitory gating of basolateral Amygdala inputs to the prefrontal cortex. J Neurosci.

[13] Jin, J, & Maren, S. Prefrontal-hippocampal interactions in memory and emotion. Front Systems Neurosci, 2015;9. 10.3389/fnsys.2015.00170. 10.3389/fnsys.2015.00170 PMC4678200 26696844

[14] Tejeda HA, Counotte DS, Oh E, Ramamoorthy S, Schultz-Kuszak KN, Bäckman CM (2013). Prefrontal cortical kappa-opioid receptor modulation of local neurotransmission and conditioned place aversion. Neuropsychopharmacology.

[15] George O, Sanders C, Freiling J, Grigoryan E, Vu S, Allen CD (2012). Recruitment of medial prefrontal cortex neurons during alcohol withdrawal predicts cognitive impairment and excessive alcohol drinking. Proc Natl Acad Sci USA.

[16] Robinson SL, Perez-Heydrich CA, Thiele TE (2019). Corticotropin releasing factor type 1 and 2 receptor signaling in the medial prefrontal cortex modulates Binge-like ethanol consumption in C57BL/6J mice. Brain Sci.

[17] Robinson SL, Marrero IM, Perez-Heydrich CA, Sepulveda-Orengo MT, Reissner KJ, Thiele TE (2019). Medial prefrontal cortex neuropeptide Y modulates binge-like ethanol consumption in C57BL/6J mice. Neuropsychopharmacology.

[18] Urban-Ciecko J, Barth AL (2016). Somatostatin-expressing neurons in cortical networks. Nat Rev Neurosci.

[19] Brockway, DF, & Crowley, NA. Turning the ′Tides on neuropsychiatric diseases: the role of peptides in the prefrontal cortex. Front Behav Neurosci, 2020;14. 10.3389/fnbeh.2020.588400 PMC7606924 33192369

[20] Crowley NA, Magee SN, Feng M, Jefferson SJ, Morris CJ, Dao NC (2019). Ketamine normalizes binge drinking-induced defects in glutamatergic synaptic transmission and ethanol drinking behavior in female but not male mice. Neuropharmacology.

[21] Pleil KE, Lowery-Gionta EG, Crowley NA, Li C, Marcinkiewcz CA, Rose JH (2015). Effects of chronic ethanol exposure on neuronal function in the prefrontal cortex and extended amygdala. Neuropharmacology.

[22] Varodayan FP, Sidhu H, Kreifeldt M, Roberto M, Contet C (2018). Morphological and functional evidence of increased excitatory signaling in the prelimbic cortex during ethanol withdrawal. Neuropharmacology.

[23] Fee C, Banasr M, Sibille E (2017). Somatostatin-positive gamma-aminobutyric acid interneuron deficits in depression: cortical microcircuit and therapeutic perspectives. Biol Psychiatry.

[24] Fuchs T, Jefferson SJ, Hooper A, Yee PH, Maguire J, Luscher B (2017). Disinhibition of somatostatin-positive GABAergic interneurons results in an anxiolytic and antidepressant-like brain state. Mol Psychiatry.

[25] Soumier A, Sibille E (2014). Opposing effects of acute versus chronic blockade of frontal cortex somatostatin-positive inhibitory neurons on behavioral emotionality in mice. Neuropsychopharmacology.

[26] Cummings KA, Clem RL (2020). Prefrontal somatostatin interneurons encode fear memory. Nat Neurosci.

[27] Xu H, Liu L, Tian Y, Wang J, Li J, Zheng J (2019). A disinhibitory microcircuit mediates conditioned social fear in the prefrontal cortex. Neuron.

[28] Rhodes JS, Best K, Belknap JK, Finn DA, Crabbe JC (2005). Evaluation of a simple model of ethanol drinking to intoxication in C57BL/6J mice. Physiol Behav.

[29] Rinker JA, Marshall SA, Mazzone CM, Lowery-Gionta EG, Gulati V, Pleil KE (2017). Extended amygdala to ventral tegmental area corticotropin-releasing factor circuit controls binge ethanol intake. Biol Psychiatry.

[30] Dao, NC, Suresh Nair, M, Magee, SN, Moyer, JB, Sendao, V, Brockway, DF, et al. Forced abstinence from alcohol induces sex-specific depression-like behavioral and neural adaptations in somatostatin neurons in cortical and amygdalar regions. Front Behav Neurosci. 2020;14. 10.3389/fnbeh.2020.00086. 10.3389/fnbeh.2020.00086 PMC7266989 32536856

[31] Vardy E, Robinson JE, Li C, Olsen RHJ, DiBerto JF, Giguere PM (2015). A new DREADD facilitates the multiplexed chemogenetic interrogation of behavior. Neuron.

[32] Jiang, C, Wang, X, Le, Q, Liu, P, Liu, C, Wang, Z, et al. Morphine coordinates SST and PV interneurons in the prelimbic cortex to disinhibit pyramidal neurons and enhance reward. Mol Psychiatry. 2019. 10.1038/s41380-019-0480-7. 10.1038/s41380-019-0480-7 PMC7985023 31413370

[33] Dao NC, Brockway DF, Crowley NA (2019). In vitro optogenetic characterization of neuropeptide release from prefrontal cortical somatostatin neurons. Neuroscience.

[34] Kroener, S, Mulholland, PJ, New, NN, Gass, JT, Becker, HC, & Chandler, LJ. Chronic alcohol exposure alters behavioral and synaptic plasticity of the rodent prefrontal cortex. PLoS ONE. 2012;7. 10.1371/journal.pone.0037541. 10.1371/journal.pone.0037541 PMC3364267 22666364

[35] Hughes BA, Bohnsack JP, O’Buckley TK, Herman MA, Morrow AL (2019). Chronic ethanol exposure and withdrawal impair synaptic GABAA receptor-mediated neurotransmission in deep-layer prefrontal cortex. Alcohol: Clin Exp Res.

[36] Joffe ME, Winder DG, Conn PJ (2021). Increased synaptic strength and mGlu2/3 receptor plasticity on mouse prefrontal cortex intratelencephalic pyramidal cells following intermittent access to ethanol. Alcohol: Clin Exp Res.

[37] Anastasiades PG, Boada C, Carter AG (2019). Cell-type-specific D1 dopamine receptor modulation of projection neurons and interneurons in the prefrontal cortex. Cereb Cortex.

[38] Bhattacherjee A, Djekidel MN, Chen R, Chen W, Tuesta LM, Zhang Y (2019). Cell type-specific transcriptional programs in mouse prefrontal cortex during adolescence and addiction. Nat Commun.

[39] Gee S, Ellwood I, Patel T, Luongo F, Deisseroth K, Sohal VS (2012). Synaptic activity unmasks dopamine D2 receptor modulation of a specific class of layer V pyramidal neurons in prefrontal cortex. J Neurosci.

[40] Ye, L, Allen, WE, Thompson, KR, Tian, Q, Hsueh, B, Ramakrishnan, C, et al. Wiring and molecular features of prefrontal ensembles representing distinct experiences. Cell. 2016 10.1016/j.cell.2016.05.010. 10.1016/j.cell.2016.05.010 PMC5708551 27238022

[41] Otis JM, Namboodiri VMK, Matan AM, Voets ES, Mohorn EP, Kosyk O (2017). Prefrontal cortex output circuits guide reward seeking through divergent cue encoding. Nature.

[42] Vander Weele CM, Siciliano CA, Matthews GA, Namburi P, Izadmehr EM, Espinel IC (2018). Dopamine enhances signal-to-noise ratio in cortical-brainstem encoding of aversive stimuli. Nature.

[43] McFarland, K, Lapish, CC, & Kalivas, PW. Prefrontal glutamate release into the core of the nucleus accumbens mediates cocaine-induced reinstatement of drug-seeking behavior. J Neurosci. 2003. 10.1523/jneurosci.23-08-03531.2003. 10.1523/JNEUROSCI.23-08-03531.2003 PMC6742291 12716962

[44] McGlinchey EM, James MH, Mahler SV, Pantazis C, Aston-Jones G. Prelimbic to accumbens core pathway is recruited in a dopamine-dependent manner to drive cued reinstatement of cocaine seeking. J Neurosci. 2016;36:8700. 10.1523/JNEUROSCI.1291-15.2016 PMC4987439 27535915

[45] Siciliano CA, Noamany H, Chang CJ, Brown AR, Chen X, Leible D (2019). A cortical-brainstem circuit predicts and governs compulsive alcohol drinking. Science.

[46] Joffe ME, Winder DG, Conn PJ (2020). Contrasting sex-dependent adaptations to synaptic physiology and membrane properties of prefrontal cortex interneuron subtypes in a mouse model of binge drinking. Neuropharmacology.

[47] Penzo MA, Robert V, Li B (2014). Fear conditioning potentiates synaptic transmission onto long-range projection neurons in the lateral subdivision of central amygdala. J Neurosci.

[48] Fuchs RA, Evans KA, Ledford CC, Parker MP, Case JM, Mehta RH (2005). The role of the dorsomedial prefrontal cortex, basolateral amygdala, and dorsal hippocampus in contextual reinstatement of cocaine seeking in rats. Neuropsychopharmacology.

[49] Marcus, DJ, Bedse, G, Gaulden, A, Ryan, JD, Clauss, J, Delpire, E, et al. Endocannabinoid signaling collapse mediates stress-induced amygdalo-cortical strengthening. Neuron, 2019;105:1062. 10.1016/j.neuron.2019.12.024. 10.1016/j.neuron.2019.12.024 PMC7992313 31948734

[50] Gass, JT, & Chandler, LJ. The plasticity of extinction: contribution of the prefrontal cortex in treating addiction through inhibitory learning. Front Psychiatry, 2013;4. 10.3389/fpsyt.2013.00046. 10.3389/fpsyt.2013.00046 PMC3667556 23750137

[51] Pati D, Pina MM, Kash TL (2019). Ethanol-induced conditioned place preference and aversion differentially alter plasticity in the bed nucleus of stria terminalis. Neuropsychopharmacology.

[52] Varodayan FP, De Guglielmo G, Logrip ML, George O, Roberto M (2017). Alcohol dependence disrupts amygdalar L-type voltage-gated calcium channel mechanisms. J Neurosci.

[53] Salling MC, Skelly MJ, Avegno E, Regan S, Zeric T, Nichols E (2018). Alcohol consumption during adolescence in a mouse model of binge drinking alters the intrinsic excitability and function of the prefrontal cortex through a reduction in the hyperpolarization-activated cation current. J Neurosci.

[54] Gu N, Vervaeke K, Hu H, Storm JF (2005). Kv7/KCNQ/M and HCN/h, but not KCa2/SK channels, contribute to the somatic medium after-hyperpolarization and excitability control in CA1 hippocampal pyramidal cells. J Physiol.

[55] Sparta DR, Hovelsø N, Mason AO, Kantak PA, Ung RL, Decot HK (2014). Activation of prefrontal cortical parvalbumin interneurons facilitates extinction of reward-seeking behavior. J Neurosci.

[56] Mazzone CM, Pati D, Michaelides M, DiBerto J, Fox JH, Tipton G (2018). Acute engagement of Gq-mediated signaling in the bed nucleus of the stria terminalis induces anxiety-like behavior. Mol Psychiatry.

[57] McElligott ZA, Winder DG (2008). α1-Adrenergic receptor-induced heterosynaptic long-term depression in the bed nucleus of the stria terminalis is disrupted in mouse models of affective disorders. Neuropsychopharmacology.

[58] Grueter BA, Gosnell HB, Olsen CM, Schramm-Sapyta NL, Nekrasova T, Landreth GE (2006). Extracellular-signal regulated kinase 1-dependent metabotropic glutamate receptor 5-induced long-term depression in the bed nucleus of the stria terminalis is disrupted by cocaine administration. J Neurosci.

[59] Colucci M, Maione F, Bonito MC, Piscopo A, Di Giannuario A, Pieretti S (2008). New insights of dimethyl sulphoxide effects (DMSO) on experimental in vivo models of nociception and inflammation. Pharmacol Res.

