# Supplementary material for: Somatostatin neurons control an alcohol binge drinking prelimbic microcircuit in mice
Source: Neuropsychopharmacology. 2021 Jun 10;46(11):1906–17. doi: 10.1038/s41386-021-01050-1 (PMC8429551; doi:10.1038/s41386-021-01050-1)
Supplement: Supplementary file 1 — Supplementary Material [file 41386_2021_1050_MOESM1_ESM.pdf]

SUPPLEMENTAL MATERIALS

Supplemental Methods

Animals

All mice were single-housed (vivarium temperature 21°C, ±1°C) on a 12 hr reverse light cycle (lights off at 7:00 am) at least one week before experimental manipulation and for the duration of the experiments, as is consistent with choice alcohol consumption paradigms (28,29). Mice had *ad libitum* access to food and water (except for during the DID procedure, described below, when water was removed). Neither gonadal hormones (estrogen or testosterone) nor estrus cycles were tracked.

Surgeries

The syringe was left in place for an extra 5 minutes before being slowly removed to limit efflux of virus. Bupivacaine (4 mg/kg) and ketoprofen (5 mg/kg) were applied topically and intraperitoneally, respectively, for postoperative pain management. Following surgery, mice were monitored and allowed to recover for one week. At least 3 weeks elapsed between the time of viral injection and experimental manipulations (Designer Receptors Exclusively Activated by Designer Drugs, DREADD; ligands administration or electrophysiology) to allow for ample viral expression.

Viral Vectors

AAV5-hSyn-DIO-mCherry, AAV8-hSyn-DIO-hM3D(Gq)-mCherry, AAV8-hSyn-dF-HA-KORD-IRES-mCitrine, AAV5-EF1a-dF-hChR2(H134R)-eYFP-WPRE-HGHpA, AAV5-CaMKIIa-hChR2(H134R)-EYFP, and AAV5-CaMKIIa-hM4D(Gi)-mCherry, described elsewhere (30,31,32), were obtained from Addgene (Watertown, MA).

Drug Administration

Mice were habituated to handling for injections for three consecutive days before the first vehicle injection. For EtOH DID, mice received vehicle injections of either 1% DMSO in saline (10 ml/kg; intraperitoneal, i.p.) or pure DMSO (1 ml/kg; subcutaneously, s.c.) on cycle 2. Injections of CNO and SalB were counterbalanced across mice to account for any order effects (i.e., mice received either CNO (3 mg/kg; i.p.) or SalB (10 mg/kg; s.c.) on cycle 3, and the other drug on cycle 4). For sucrose DID, mice received vehicle injections on cycle 1, and CNO or SalB counterbalanced injections on cycle 2 or 3. For the hM4D-mediated inhibition of pyramidal neurons experiment, mice were administered saline on cycle 2 and CNO (3 mg/kg) on cycle 3. All injections were done 30-40 minutes before the start of the binge session (30).

General electrophysiology

Mice were deeply anesthetized via inhaled isoflurane and rapidly decapitated. Brains were quickly removed and processed according to the NMDG protective recovery method (35). Briefly, brains were immediately placed in ice-cold oxygenated N-methyl-D-glucamine (NMDG)-HEPES aCSF containing the following, in mM: 92 NMDG, 2.5 KCl, 1.25 NaH_2PO_4, 30 $NaHCO_3$, 20 HEPES, 25 glucose, 2 thiourea, 5 Na-ascorbate, 3 Na-pyruvate, 0.5 $CaCl_2 \cdot 2H_2O$, and 10 $MgSO_4 \cdot 7H_2O$ (pH to 7.3–7.4). The PL was identified according to the Allen Mouse Brain Atlas. 300 μM coronal slices containing the PL were prepared on a Compresstome vibrating microtome VF-300-0Z (Precisionary Instruments, Greenville, NC), and transferred to heated (31°C) NMDG-HEPES aCSF for a maximum of 10 min. Slices were then transferred to heated (31°C) oxygenated normal aCSF (in mM: 124 NaCl, 4.4 KCl, 2 $CaCl_2$, 1.2 $MgSO_4$, 1 NaH_2PO_4, 10.0 glucose, and 26.0 $NaHCO_3$, pH 7.4, mOsm 300-310), where they were allowed to rest for at least 1 h before use. Finally, slices were moved to a submerged recording chamber (Warner Instruments, Hamden, CT) where they were continuously perfused with the recording aCSF (2 ml per min flow rate,

31°C). Recording electrodes (3–6 MΩ) were pulled from thin-walled borosilicate glass capillaries with a Narishige P-100 Puller.

Pyramidal neurons in layer 2/3 of the PL cortex were identified by location, morphology (prominent triangular soma and apical dendrites), and membrane characteristics (capacitance > 75 pF, or membrane resistance < 100 mΩ). SST-expressing neurons were identified in SST-IRES-Cre::Ai9 mice via presence of tdTomato fluorescence under a 40x immersed objective with 565 nm LED excitation. Non-SST expressing neurons (putatively other GABAergic neurons) were identified by lack of fluorescence, morphology, and membrane characteristics (low capacitance, and membrane resistance > 200 mΩ).

SST optogenetic electrophysiology

Neurons were held at -50 mV, and photostimulation of SST neurons produced outward currents onto pyramidal neurons and non-SST neurons that were sensitive to blockage by the $GABA_A$ receptor antagonist picrotoxin (100 µM; **Supplementary Figure 2A**). Evoked IPSC peak amplitude induced by the first LED pulse and paired-pulse ratio (PPR: amplitude of pulse 2/amplitude of pulse 1) were measured as proxies of synaptic strength between SST neurons and other neuronal populations in the PL cortex. Photostimulations were performed across multiple laser intensities (10%-100%, 10% interval), however the peak optically-evoked IPSC amplitude did not significantly vary with laser intensity (data not shown) so only data derived from maximum laser intensity are reported. The cell type of recorded neurons was then confirmed in current clamp mode, in which action potential properties were assessed via a V-I protocol (0-300 pA, 10 pA per step). To confirm monosynaptic connections between PL SST neurons and other populations, TTX (500 nM) and 4-aminopyridine (4-AP, 100 µM) was bath applied for 10 min after 5 min of stable optically evoked IPSC to eliminate polysynaptic currents (**Supplementary Figure 3**).

Histology

Mice were deeply anesthetized with Avertin (250 mg/kg), and transcardially perfused with ice-cold phosphate buffered saline (PBS) then 4% (w/v) paraformaldehyde (PFA). Brains were post-fixed in PFA overnight and sectioned at 40 microns using a Compresstome vibrating microtome. PL cortex-containing sections were incubated in 1:20,000 DAPI in PBS for 30 min, mounted on SuperFrost glass slides, air-dried, and coverslipped with ImmunoMount mounting media. Viral injections were assessed under mCherry and/or mCitrine fluorescence filter in an Olympus BX63 upright microscope (Center Valley, PA), and the brightest fluorescence point was chosen as the center of the injection. Mice with unilateral viral expression, and/or missed injections (including expression in the infralimbic cortex) were excluded from analysis (n = 4 for hM3Dq/KORD DREADD experiment, n = 1 for SST-opto evoked IPSC experiment, n = 2 for CamKIIa-hM4Di experiment). Quantification of overall injection localization was performed in ImageJ (NIH) and presented in **Figure 2B** and **Supplementary Figure 2**. Cells visualizable with both mCherry and mCitrine fluorescence were counted as mCitrine+/mCherry+, whereas those visualizable with either mCherry or mCitrine only were counted as mCherry+ or mCitrine+, respectively.

Supplemental Figures

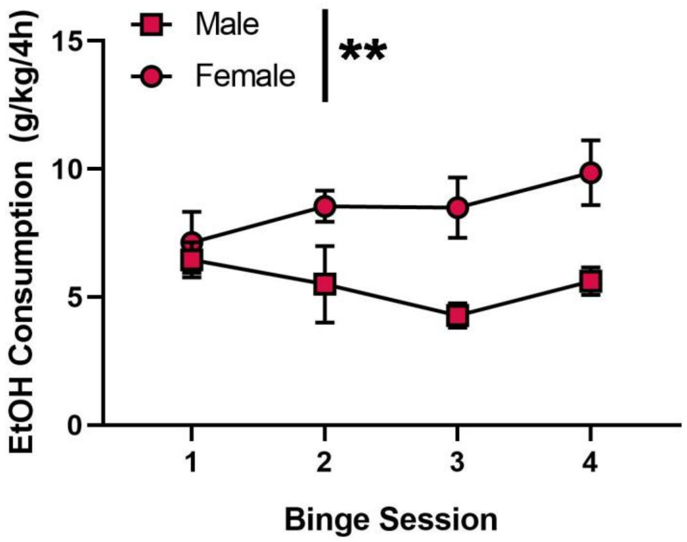

Supplementary Figure 1. Female mice engage in higher level of alcohol binge drinking than male mice, related to Figure 1. Average consumption of 20% (v/v) ethanol solution in 4-h binge session in Drinking-in-the-Dark (DID) paradigm showed a sex difference (N = 6 males and 5 females, $F_{sex}(1,9) = 15.07$, $p = 0.003$), and these levels are stable across cycles ($F_{cycle}(3,27) = 0.713$, $p = 0.552$, $F_{sex\ x\ binge}(3,27) = 1.561$, $p = 0.221$)

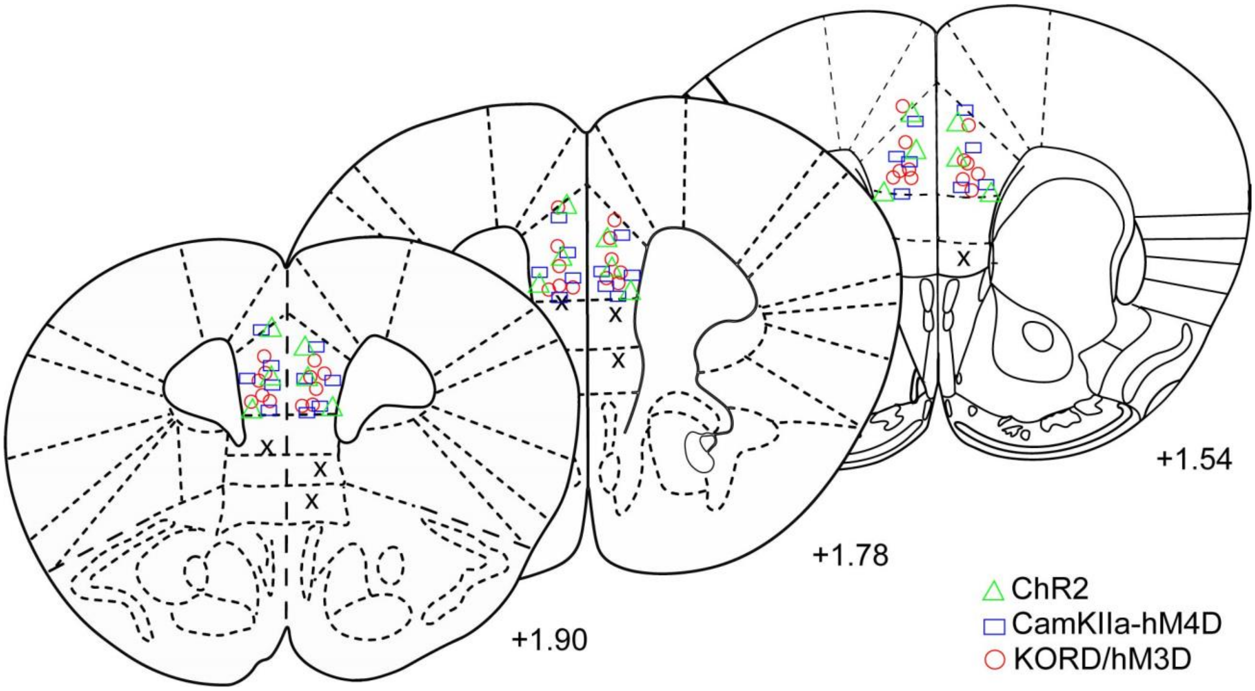

Supplementary Figure 2. Histological verification of viral expression in the PL, related to figures 2, 3 and 4.

Each symbol represents the center of injections, where the expression of fluorophore-tagged virus is the brightest. Red circles: KORD/hM3Dq mice. Green triangles: ChR2 mice. Blue rectangles: CamKIIa promoter-hM4D mice. X: missed injection.

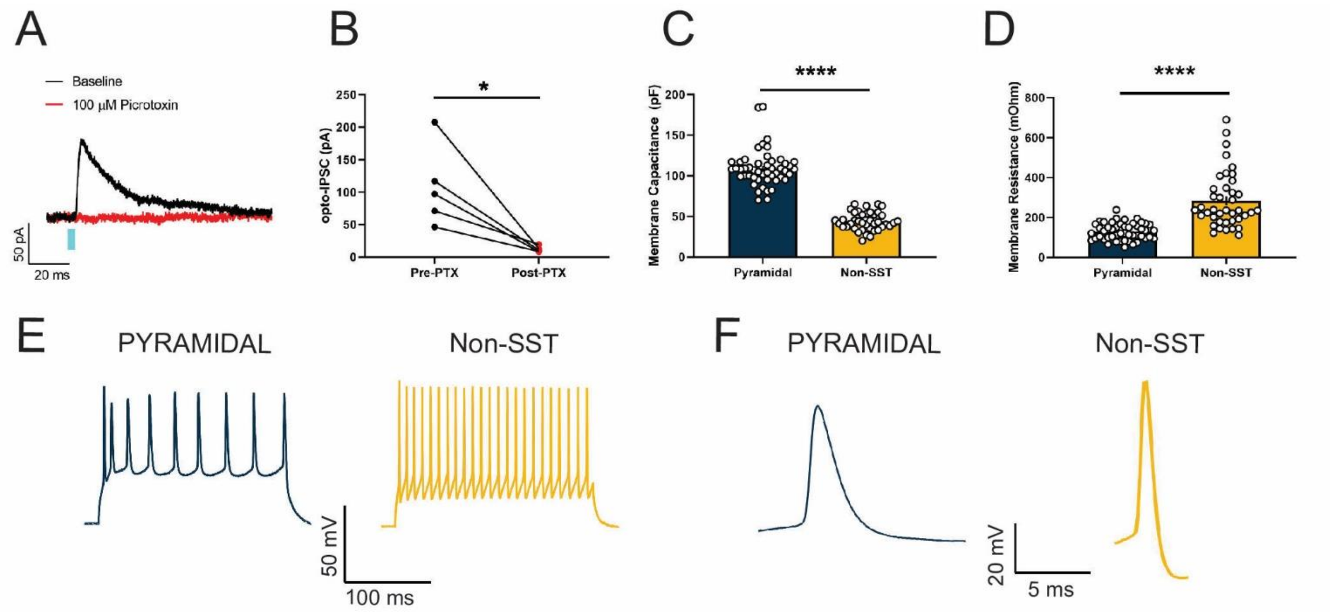

Supplementary Figure 3. Membrane properties of pyramidal neurons and non-SST neurons in the PL, related to Figure 3.

(A) Representative traces of outward currents in pyramidal neurons in the PL following photostimulation of SST neurons during baseline (black) and after bath application of the $GABA_A$ receptor antagonist picrotoxin.

(B) Bath application of 100 µM picrotoxin completely abolished SST-evoked IPSC in pyramidal neurons (paired $t(4) = 3.465$, $p = 0.025$).

(C) Pyramidal neurons in the PL exhibit distinctively higher membrane capacitance than non-SST neurons (unpaired $t(87) = 16.84$, $p < 0.001$).

(D) Pyramidal neurons in the PL exhibit lower membrane resistance than non-SST neurons (unpaired $t(87) = 7.396$, $p < 0.001$).

(E) Representative traces of action potentials in pyramidal neurons and non-SST neurons in response to a +200 pA somatic current injection at RMP.

(F) Representative single action potentials in pyramidal neurons and non-SST neurons. Cells were classified as pyramidal neurons with long action potential width (>6 ms), or as non-SST neurons with short action potential width (1-3 ms).
